# An identification method of human joint interaction torque based on discrete EMG signals

**DOI:** 10.3389/fbioe.2025.1596180

**Published:** 2025-04-29

**Authors:** Liangchuang Liao, Ding Yan, Guoan Zhang

**Affiliations:** ^1^ School of Electronic Science and Engineering, Southeast University, Nanjin, Jiangsu, China; ^2^ China Shipbuilding Digital Information Technology Co., Ltd., Beijing, China; ^3^ School of Automation, Nanjing University of Aeronautics and Astronautics, Nanjing, Jiangsu, China; ^4^ Institute of Intelligent Manufacturing, Taizhou University, Taizhou, Zhejiang, China

**Keywords:** exoskeleton robotics, electromyography signals, joint output torque, prediction, motion intention

## Abstract

**Introduction:**

The interactive joint torque serves as a critical biomechanical parameter for intent recognition in exoskeleton motion control systems, enabling adaptive control capabilities within the human-in-the-loop (HITL) closed-loop framework. While this interactive torque fundamentally differs from the actual output torque of joints, empirical studies have demonstrated a quantifiable linear correlation between these two metrics. Consequently, real-time monitoring of joint output torque provides actionable insights into human motion intention, serving as a critical feedback mechanism for intention-driven control strategies in lower-limb exoskeleton applications.

**Method:**

This paper proposes a method for extracting the interactive joint torque of the human body based on the collection of discrete electromyography (EMG) signals. In order to detect and analyze the interactive joint torque, based on the acquisition of human EMG signals, the human joint motion is discretized within a continuous range using a discrete prediction method. Then, the results of discrete learning are converted into a continuous form to establish a numerical relationship between human muscle movement and interactive joint torque.

**Result:**

This identification method has high accuracy under different motion states of the human body. The mean square error of all experiments is 0.1502, the mean coefficient of determination is 0.8616, and the mean coefficient of correlation is 0.9365.

**Discussion:**

A discrete prediction technology of human joint interaction torque based on EMG acquisition is established, which is helpful to deeply understand the relationship between muscle activity and joint motion, and provides a feasible method for extracting human joint torque.

## 1 Introduction

The wearable architecture of exoskeleton robots establishes unique control paradigms fundamentally distinct from autonomous robotic systems. Unlike conventional industrial robots, the human operator demonstrates exceptional adaptive learning capacity, autonomously optimizing movement strategies through neuromuscular adaptation. However, exoskeleton deployment creates bidirectional kinetic coupling between human and machine, forming a closed-loop biomechanical system that inherently integrates the motion intention and physiological capability of operators as real-time control inputs. This integration necessitates the quantitative characterization of human joint interactive torque – a critical biomechanical parameter requiring precise acquisition for closed-loop control implementation. Specifically, the human joint interactive torque must be systematically quantified and embedded within the control architecture to reconcile human-robot kinetic interactions (Cheng et al., 2021; Li et al., 2014; Aung and Al-Jumaily, 2013; Hayashibe et al., 2009).

In the human-exoskeleton interaction system, EMG signals can enable continuous joint motion estimation, including joint torque, angular velocity, and angle (Ding et al., 2016; Lobo-Prat et al., 2014; Oskoei and Hu, 2007). Currently, prediction methods for continuous human joint motion estimation based on EMG signals mainly fall into two categories: those based on biomechanical models and those based on machine learning. Among the biomechanical model-based methods for predicting human joint motion, Xiong B (Xiong et al., 2020) proposed an intelligent prediction method based on the Hill muscle model to determine human joint torque with online measurable input variables. This method utilizes electromyograms, joint angles, and angular velocities as inputs to predict joint torque, with higher accuracy compared to methods using other input variables. Li K (Li et al., 2019) and others used surface EMG (sEMG) signals and estimated elbow joint angles based on muscle biomechanical properties. They optimized unknown parameters using a genetic algorithm, achieving an average root mean square error ranging from 0.12 to 0.26 radians, demonstrating high estimation accuracy. While the Hill muscle model-based motion estimation model has specific physiological meanings and interpretability, many physiological parameters cannot be directly measured, and the model construction is complex, with accumulated errors. Therefore, the practicality of biomechanical model-based methods is limited and challenging to apply to the prediction of exoskeleton robot joint motion states.

Machine learning-based motion estimation prediction methods overcome the drawbacks of the aforementioned methods, such as complex models and poor prediction accuracy. Sangheum Lee (Lee et al., 2020) and others proposed a real-time joint torque estimation method using sEMG signals and artificial neural networks on an embedded system. The two-layer, three-node artificial neural network precisely maps EMG signals to target torque values, achieving determinism and reducing the reaction time of EMG signals in estimating joint torque by 15 ms compared to traditional physical sensors. Chinmay P. Swami (Swami et al., 2021) introduced a machine learning framework based on neural networks and random forests for designing a multi-degree-of-freedom prosthetic wrist controller, enabling natural control of the prosthetic wrist in daily activities. Wang C (Wang et al., 2020) and others proposed a continuous estimation method for six daily grasping movements based on the Long Short-Term Memory (LSTM) network and compared it with Sparse Gaussian Processes (SPGP) and Radial Basis Function Neural Network (RBF). Testing with the NinaPro dataset showed that LSTM had higher correlation, smaller mean squared error, and standardized mean squared error for estimating the six grasping movements. Liang J (Liang et al., 2021) presented a knee joint angle prediction model based on sEMG signals, combining Gaussian process models with muscle activation physiological characteristics. They developed a non-parametric probabilistic model to address the instability of EMG signals and uncertainty in the neuromuscular system. From the perspective of learning from EMG signals, machine learning-based prediction of human joint torque offers greater flexibility, adaptability, and personalized customization, providing more accurate and reliable predictions of human motion states.

In summary, due to the inherent challenges in accurately extracting actual biomechanical joint torque from human motion, the proposed “interactive joint torque” in this study does not represent direct physiological torque measurements. Instead, it constitutes a proportional control feedback quantity that effectively captures the variation trends of joint torque dynamics. When estimating human joint torque through EMG signals, while continuous motion data enables high-precision torque prediction for specific gait patterns through machine learning approaches, this methodology demonstrates significant limitations when applied to random or unconstrained movements.

**FIGURE 1:**
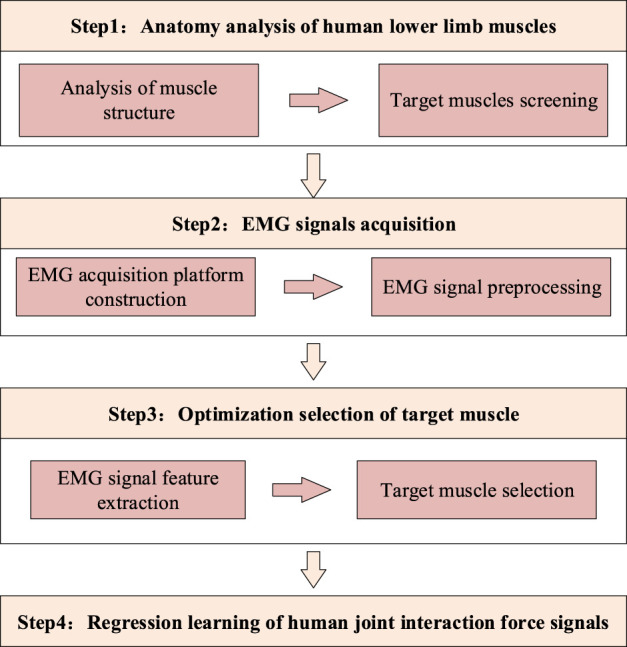
The process of human joint interaction torque identification method.

To address this constraint, a novel discrete EMG-based framework for interactive joint torque estimation is proposed, as shown in Figure 1. The proposed method implements discrete signal prediction to segment the continuous joint motion spectrum into analyzable intervals, thereby enabling effective detection and computational analysis of interactive torque components. Through subsequent transformation of discrete learning outcomes into continuous torque profiles, a quantifiable correlation between muscular activation patterns and resultant interactive joint torque is established. This mathematical relationship ultimately permits reliable detection of human motion intention across variable movement conditions.

## 2 Discrete EMG acquisition platform construction and signal preprocessing

In the context of collecting EMG signals for the target muscle groups of lower-limb exoskeleton robots, human motion is characterized by suddenness, while mechanical loads exhibit variability. Consequently, continuous signal analysis employing regression learning methods fails to yield satisfactory results. To address these challenges, this paper proposes a scheme for discretizing EMG signals, with the implementation steps structured as follows:

(1) Divide the range of motion from 20° to 160° into discrete points, with each point representing a 10° interval.
(2) Fix the joint using force sensors when testing at discrete points.
(3) Obtain the torque values for knee flexion and extension movements using joint torque sensors at the fixed joint, corresponding to the values of joint EMG signals. These values serve as the basis for regression learning data.

The advantage of this method lies in its direct reflection of the variation in joint output and EMG signals at different angles, independent of load conditions and movement states. However, a drawback is that the accuracy of discrete signals depends on the degree of subdivision of the discrete intervals. As binding constraints, such as bundling, cannot ensure complete synchronization between the human body and the testing equipment, even in a fully tightened state, there may still be slight deviations. Consequently, this fluctuation range directly affects the numerical values of discrete subdivision.

### 2.1 Discrete sEMG sensor signal acquisition platform

**FIGURE 2:**
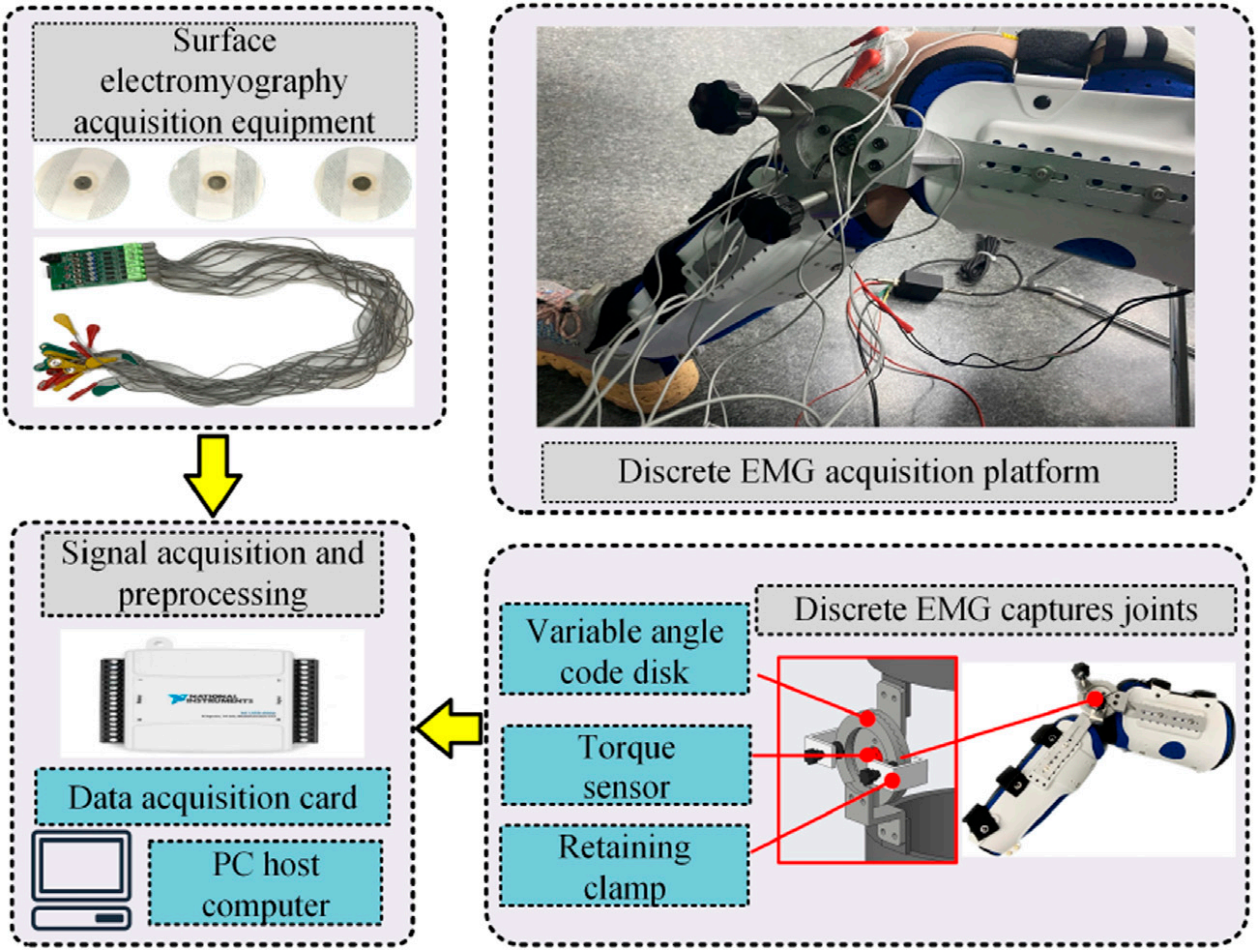
sEMG sensing signal acquisition platform.

To address the position and movement characteristics of the knee joint muscle group, a sEMG signal acquisition platform is established, as shown in Figure 2. The sEMG signal acquisition platform comprises surface electrode patches, a sc acquisition device, a knee joint angle fixation device, torque sensor, and data acquisition card.

**FIGURE 3:**
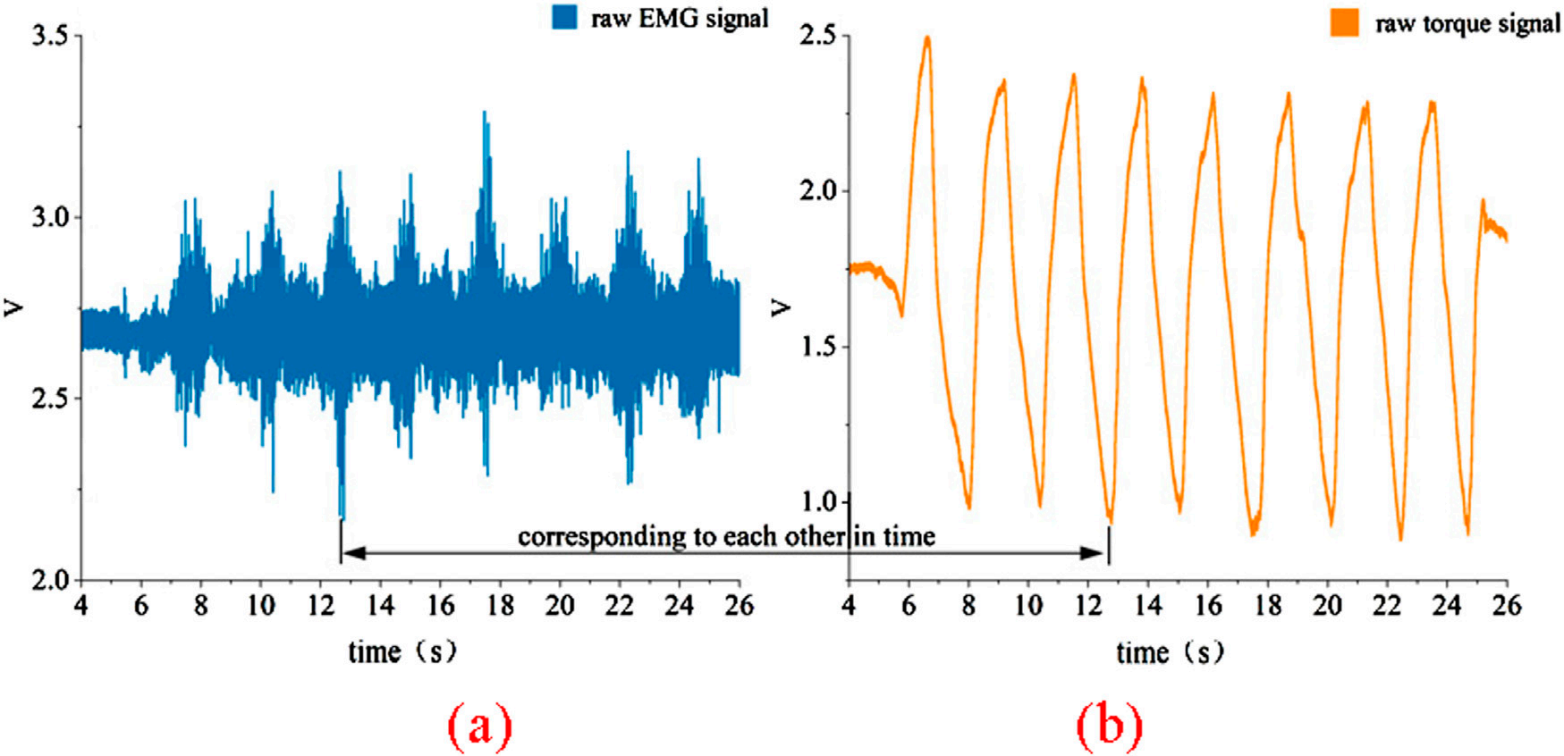
Raw data collected from platform. **(a)** Raw EMG signal **(b)** Raw torque signal.

**FIGURE 4:**
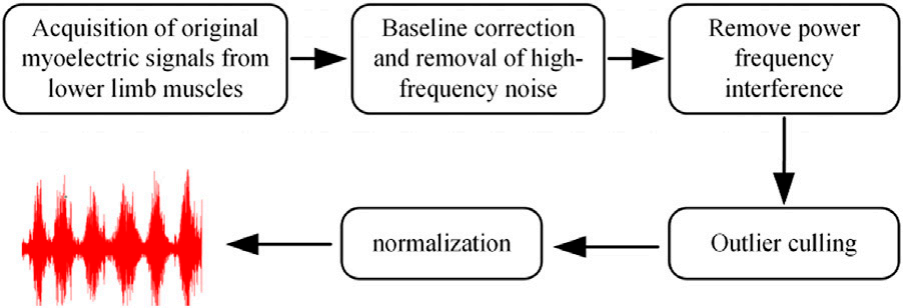
sEMG processing flowchart.

**FIGURE 5:**
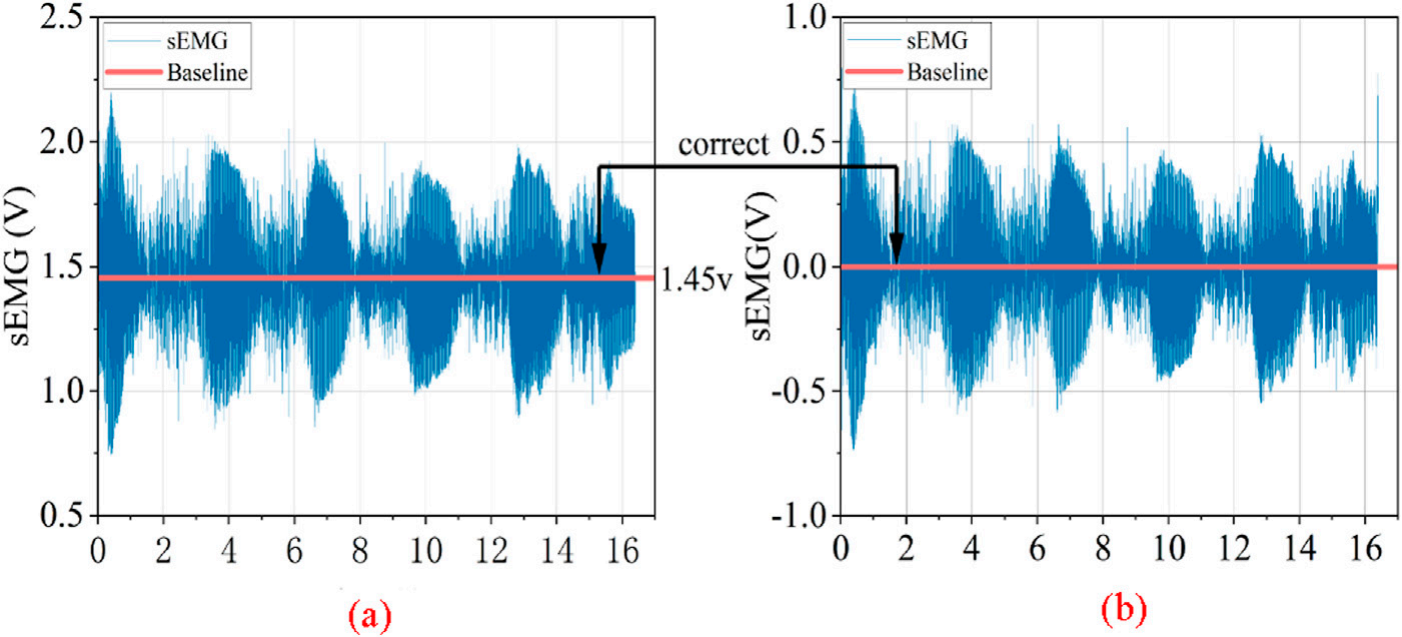
sEMG signal baseline correction. **(a)** Raw sEMG signal **(b)** Baseline corrected sEMG signal.

**FIGURE 6:**
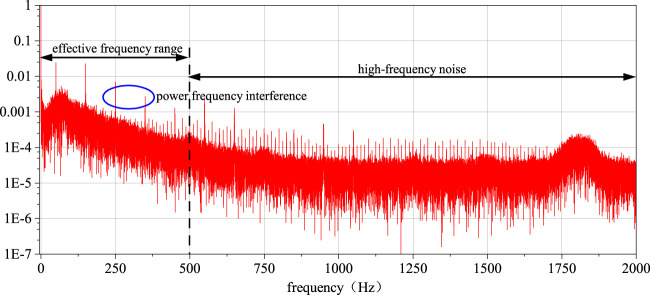
Spectrum analysis of sEMG raw signal.

**FIGURE 7:**
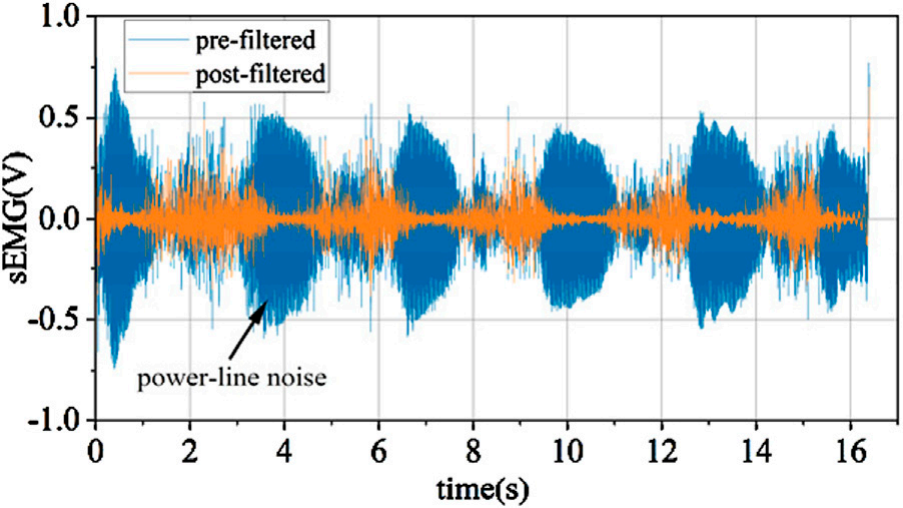
sEMG power frequency noise filtering processing.

To collect EMG signal generated by muscle exertion at different knee joint angles, the device shown in Figure 2 is employed to achieve adjustable angle fixation for the knee joint in the range of 20°–160°, as detailed in the adjustment process illustrated in Figures 3–7. The overall device is primarily composed of aluminum alloy links, an adjustable angle encoder, and straps, which can be securely fastened to the human leg.

The raw data collected using this platform is shown in Figure 3. This data was obtained by fixing the knee joint angle at 110° and performing multiple knee extension and flexion movements. The EMG signals from the quadriceps muscle and the interactive torque at the knee joint were measured using sensors on the platform. It can be observed that the raw EMG signals exhibit significant background noise, attributed to external interference and hardware-related factors, necessitating a more complex preprocessing approach. In contrast, the raw torque signals experience less interference, with minimal noise, making their preprocessing relatively straightforward.

### 2.2 sEMG signal preprocessing

In order to ensure the readability of the signals, a secondary processing of the signals was performed through software filtering, as illustrated in Figure 4.

#### 2.2.1 Correction of EMG signal baseline drift

The sEMG raw signal may exhibit baseline drift, which is related not only to the direct current component inherent in the EMG signal acquisition device but also to factors such as the decrease in skin resistance caused by sweating during the subject’s movement, leading to sweat-induced artifacts, or slow electrical activity due to electrode loosening. These factors result in a very slow (0.2–0.5 Hz) electrical activity resembling baseline drift in the sEMG signals, which falls into the category of low-frequency noise. Additionally, the sampling frequency of the raw sEMG signals is 2,000 Hz, while the effective spectral distribution of the EMG signals is between 10 and 500 Hz. Consequently, the signal also contains a significant amount of invalid high-frequency background noise (Han et al., 2015; Crouch and He, 2015; Reaz et al., 2006).

In this study, a fourth-order Butterworth band-pass filter (f1 = 10 Hz, f2 = 500 Hz) was used to address baseline drift issues, eliminate low-frequency and high-frequency noise in the EMG signals, as shown in Equation 1.

$${\left|H\left(\omega \right)\right|}^{2}=\frac{1}{1+{\left(\frac{\omega }{\omega_{c}}\right)}^{2n}}=\frac{1}{1+\epsilon^{2}{\left(\frac{\omega }{\omega_{p}}\right)}^{2n}}$$ (1)

In the formula, n represents the filter order, $\omega_{c}$ is the cutoff frequency, $\omega_{p}$ is the edge frequency of the passband, and $\frac{1}{1+\epsilon^{2}}={\left|H\left(\omega \right)\right|}^{2}$ is a numerical value at the edge of the passband. As shown in Figure 5, the baseline voltage of the EMG signal changes from 1.45 v to 0 v, which is the result after baseline correction.

#### 2.2.2 Removal of power line frequency interference in EMG signals

As depicted in Figure 6, through the analysis of the signal spectrum, it can be observed that there is a substantial amount of 50 Hz power line frequency interference and its harmonic components in the signal. Due to the power line frequency being 50 Hz, its electromagnetic noise can interfere with signal acquisition devices through wired or wireless means. The effect after eliminating power line frequency interference using multiple IIR notch filters is shown in Figure 7.

#### 2.2.3 Removal of abnormal values and normalization of EMG signals

Due to the influence of software and hardware performance, EMG signals inevitably contain more or fewer abnormal values. To prevent outliers from significantly affecting subsequent data normalization, it is necessary to remove these abnormal values. The method used in this paper for handling abnormal values is based on the Hampel identifier criterion, which removes one or more outliers that are significantly distant from other observed values in the sample. Unlike the Hampel identifier based on the judgment method of the original sample’s distance from the sample mean exceeding a certain standard value, this paper uses the sample median instead of the sample mean, effectively avoiding the influence of abnormal values on mean calculations.

**FIGURE 8:**
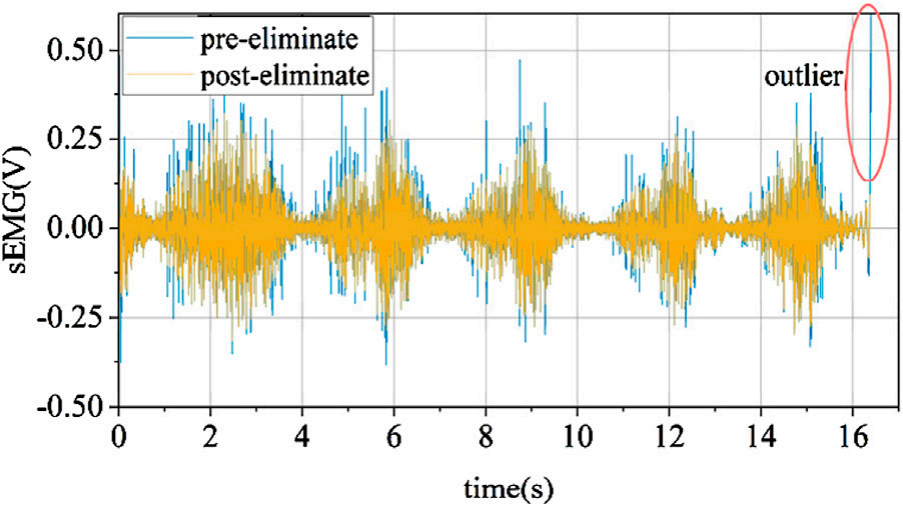
sEMG signal outlier removal processing.

This paper adopts a stepwise data storage window to extract real-time EMG signals for abnormal value removal. Each window extracts 750 data points as the total sample, calculates the median and standard deviation, and iterates over each data point in the sample. When a data point is found to have an absolute difference from the median greater than (k = 3) times the standard deviation, it is considered an abnormal value. The effect before and after removal is shown in Figure 8.

**FIGURE 9:**
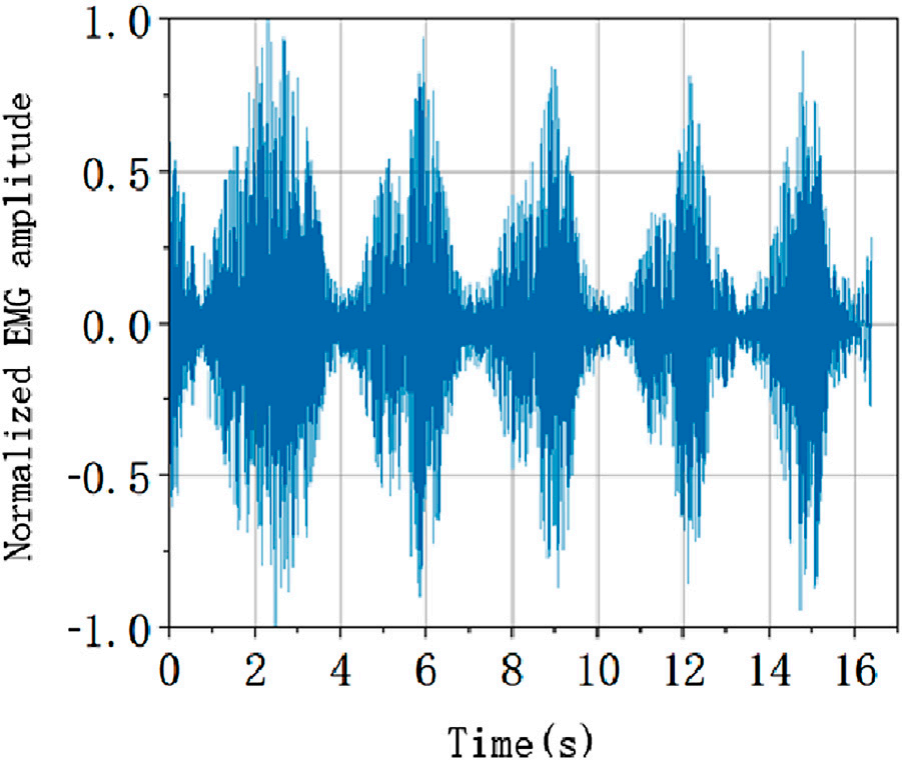
sEMG normalization processing.

It is necessary to normalize the EMG signal in order to unify each characteristic level. According to the following formula, the amplitude of the data after the elimination of outliers is linearly normalized, and the EMG amplitude is evenly distributed between −1 and 1. The normalized EMG amplitude is shown in Figure 9.

## 3 EMG signal feature extraction and muscle optimization

In order to eliminate the redundant information in the EMG signal as much as possible, it is necessary to extract the feature of the pre-processed EMG signal and re-select the muscle after the muscle primary. This section will introduce the methods of EMG feature extraction and muscle optimization.

### 3.1 EMG feature extraction

EMG signals are a type of random signal, typically exhibiting characteristics of nonlinearity, non-stationarity, and time-varying behavior. In this study, the overlapping window analysis method was applied to process the EMG signals. Parameters were set with a window length of 400 sampling points (i.e., 200 milliseconds), a window shift of 100 sampling points (i.e., 50 milliseconds), and an overlap rate of 75%. The number of features for each muscle corresponds to the number of overlapping windows. In terms of feature selection, time-domain features have lower complexity compared to other features, making them widely applicable in real-time systems, classification models, and regression models.

**FIGURE 10:**
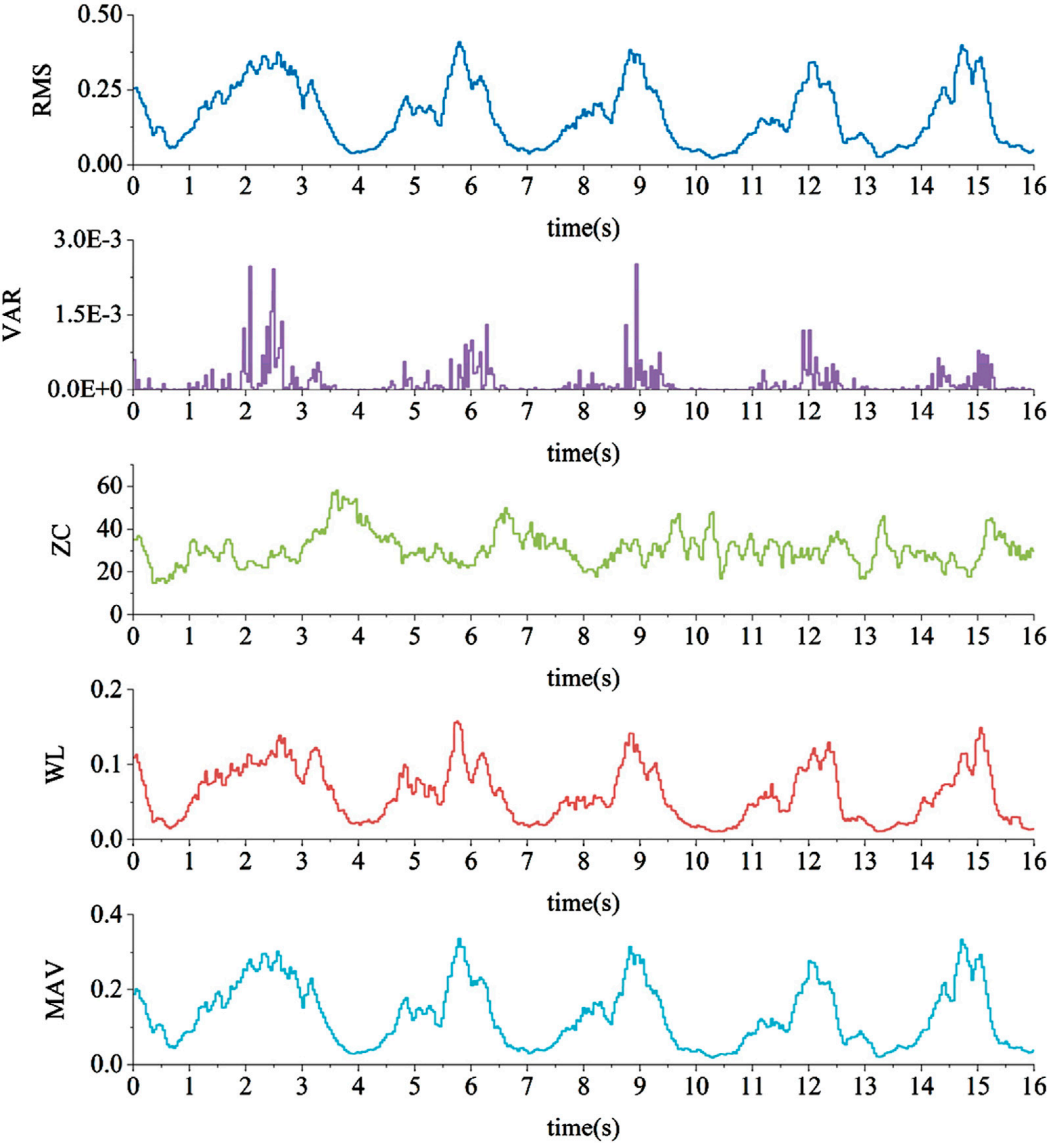
sEMG characteristic values.

This study initially selected five time-domain features for computation, namely, Root Mean Square (RMS), Variance (VAR), Zero Crossing Count (ZC), Wavelength (WL), and Mean Absolute Value (MAV). These features were calculated for each overlapping window of sEMG data obtained from the previous step. The resulting feature vectors will be used for the training and testing of the classification model. After preprocessing, the quadriceps muscle EMG signals is extracted with five features, as illustrated in Figure 10.

### 3.2 EMG feature screening

After feature extraction, it is necessary to employ feature selection methods to eliminate redundant features and select the most relevant ones for the prediction task. This is crucial for reducing redundancy and data dimensionality among features, thereby improving prediction accuracy. Correlation refers to the degree of association between two variables, and features with low correlation are involved in the subsequent training of joint torque regression models. This can lead to model overfitting; hence, it is necessary to conduct feature selection on the five features initially selected in the previous section, removing those with lower correlation.

Three extensor muscles were selected: Rectus Femoris (Ch.1), Vastus Medialis (Ch.2), and Vastus Lateralis (Ch.3), along with three flexor muscles: Biceps Femoris Long Head (Ch.4), Semitendinosus (Ch.5), and Gastrocnemius (Ch.6), forming the initial six-channel input signals. The Pearson correlation coefficient was then used to measure the correlation between the same feature across different muscles and the joint interactive torque, as shown in Equation 2.

$$corr=\frac{\sum_{i=1}^{n}\left(Fe_{i}-\bar{Fe}\right)\left(M_{i}-\bar{M}\right)}{\sqrt{\sum_{i=1}^{n}{\left(Fe_{i}-\bar{Fe}\right)}^{2}}\sqrt{\sum_{i=1}^{n}{\left(M_{i}-\bar{M}\right)}^{2}}}$$ (2)

In the formula, $Fe_{i}$ is eigenvalue, ${\bar{F}}_{e}$ is the eigenvalue means, $M_{i}$ is the joint moment, $\bar{M}$ mean joint moment, n is Sample size

The participants wore a static torque collection platform with the knee joint bending at an angle of 110°. To account for the differences in the major muscle groups involved in knee extension and knee flexion experiments, two sets of experiments were conducted. In the knee extension experiment, participants collected torque data and muscle EMG signals for five instances of knee extension movements. A correlation analysis was then performed, yielding correlation coefficients between each muscle’s various features and the joint interactive torque, as shown in Table 1. Similarly, in the knee flexion experiment, participants collected torque data and muscle EMG signals for five instances of knee flexion movements. Again, a correlation analysis was conducted, resulting in the correlation coefficients presented in Table 2. Due to the different torque directions in the knee extension and knee flexion experiments, the correlation coefficients obtained in the two sets of experiments have opposite positive and negative signs.

**TABLE 1:** Correlation coefficient between various characteristics of knee extensor muscles and joint torque.

Passage	RMS	VAR	ZC	WL	MAV
Ch.1	0.8612	0.6235	−0.2161	0.8731	0.8523
Ch.2	0.8232	0.5926	−0.3226	0.8442	0.8114
Ch.3	0.8945	0.6353	−0.3923	0.9054	0.8822
Mean	0.8596	0.6171	−0.3103	0.8742	0.8486

**TABLE 2:** Correlation coefficient between various characteristics of knee flexion muscles and joint torque.

Passage	RMS	VAR	ZC	WL	MAV
Ch.4	−0.9253	−0.6754	0.3779	−0.9334	−0.9134
Ch.5	−0.9026	−0.6364	0.2794	−0.9259	−0.9076
Ch.6	−0.8873	−0.5924	0.2235	−0.9032	−0.8721
Mean	−0.9050	−0.6347	0.2936	−0.9208	−0.8977

**FIGURE 11:**
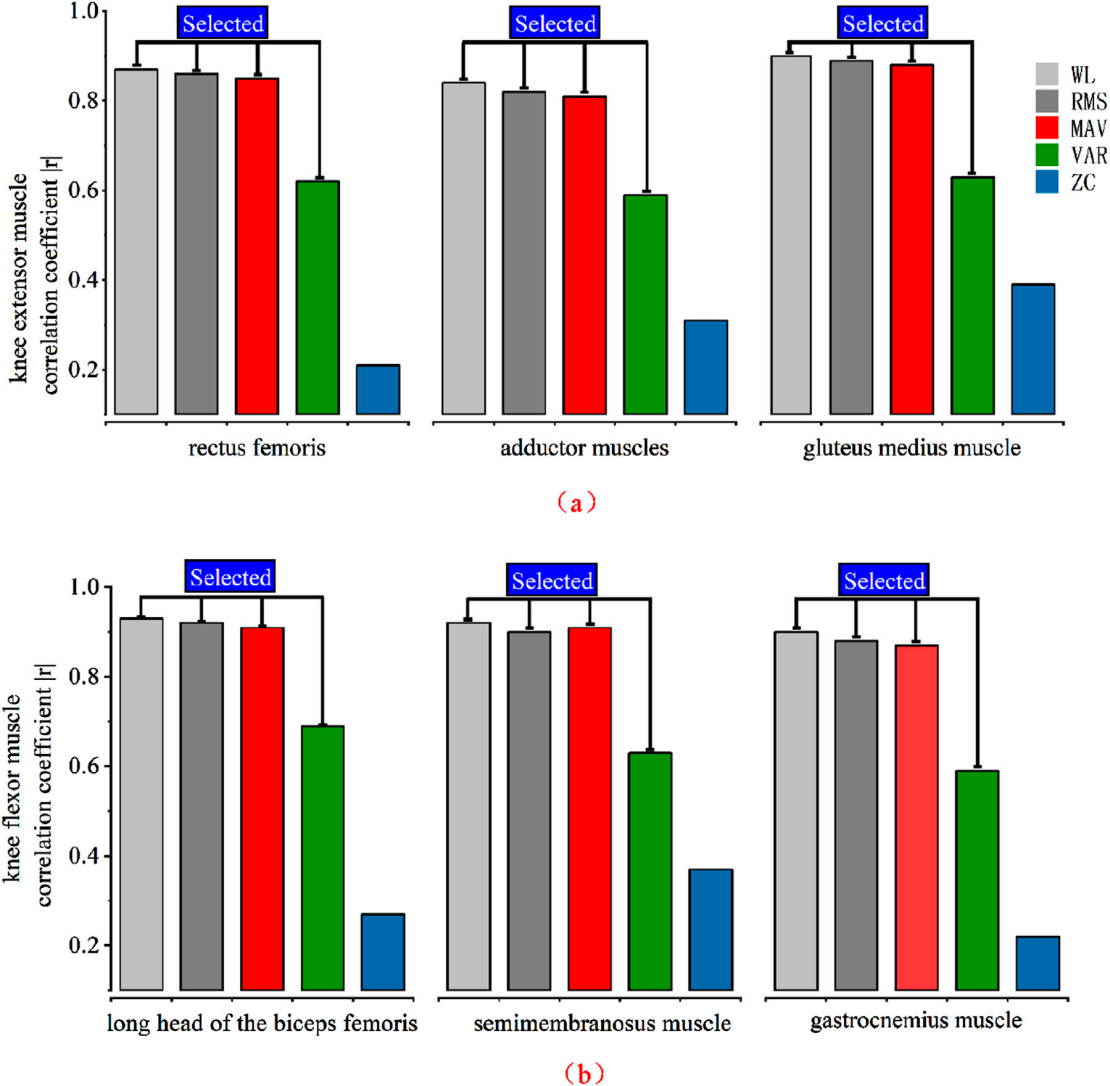
Correlation analysis of various muscles. **(a)** Knee extensor muscle feature selection **(b)** Knee flexor muscle feature selection.

The data above has been processed with absolute values and arranged in the form of a bar chart, as shown in Figure 11. It is evident that in both knee extension and knee flexion muscles, WL, RMS, and MAV exhibit a strong correlation with joint torque, while VAR shows a moderate correlation, and ZC has a lower correlation. Therefore, the feature with the lowest correlation, ZC, is removed, and RMS, VAR, WL, and MAV are retained as the final EMG signal features for use.

### 3.3 Target muscle selection

In the process of normal human walking, going up and down stairs, and squatting, knee extension or flexion movements are constantly occurring. However, considering the application scenarios of exoskeleton robots, the assistance phase mainly occurs during the human knee extension, with minimal force following during knee flexion. Therefore, to some extent, the mapping regression model of EMG signals to joint torque in the positive direction (with joint torque in the direction of knee extension considered positive and in the direction of knee flexion considered negative) becomes more important. Consequently, the number of muscles for knee flexion can be fewer than the number for knee extension.

**FIGURE 12:**
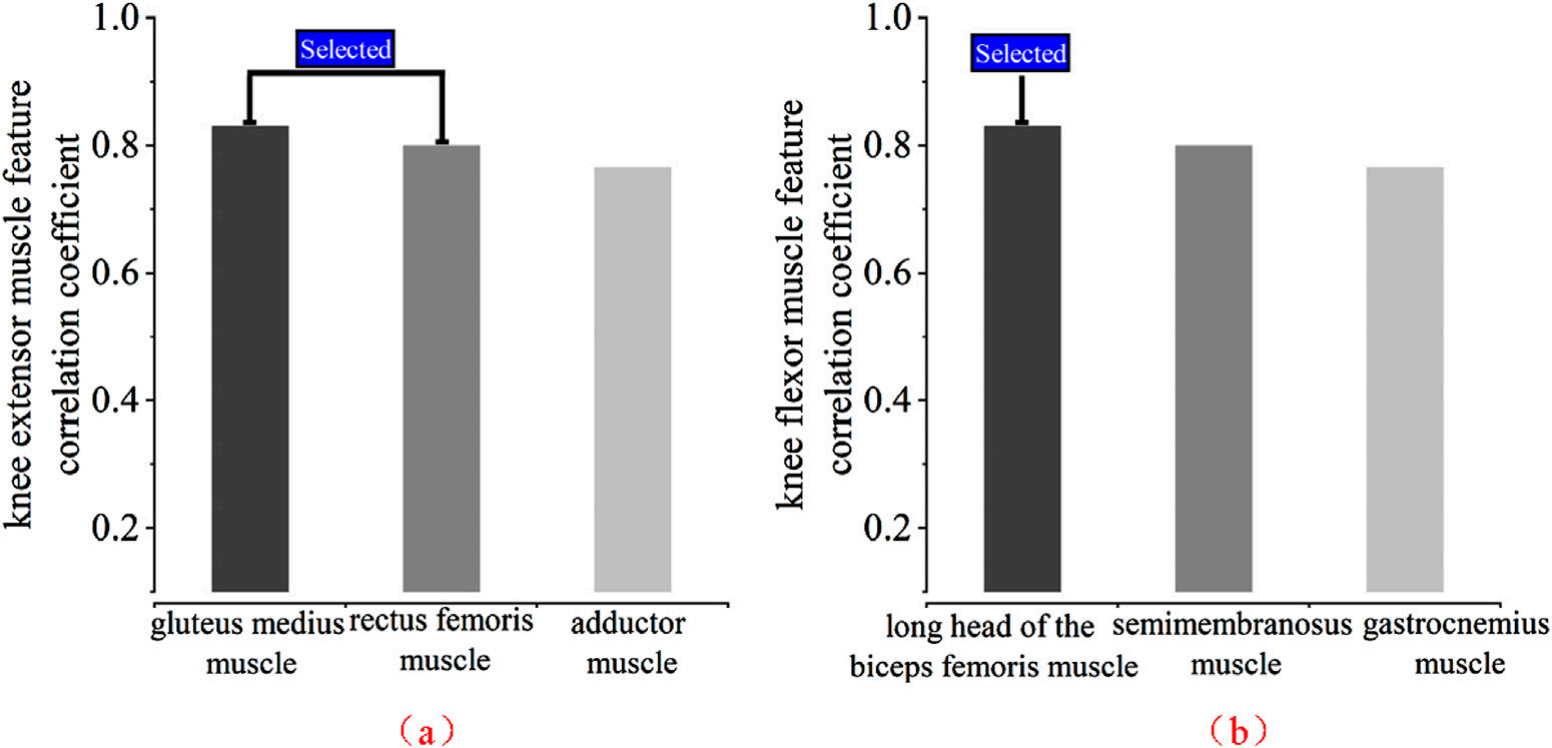
Absolute average absolute correlation coefficient of each muscle. **(a)** Knee extensor muscle selection **(b)** Knee flexor muscle selection.

Based on the features (RMS, VAR, WL, MAV) data for each muscle in Tables 1, 2, the absolute values are summed and then averaged. When grouped by knee extension and knee flexion muscles and arranged from high to low, as shown in Figure 12, the muscles gastrocnemius lateralis, vastus lateralis, and biceps femoris long head can be selected as the final muscle channels, considering the previous analysis.

### 3.4 PCAD dimension reduction

In order to further reduce the complexity of the calculation, the principal component analysis algorithm (PCA) is used to complete the feature dimensionality reduction operation. According to Equation 3, the information contained in all principal components can be measured by their variance (VAR). The greater the variance VAR(Pci), the more information contained in the component, and the first principal component usually contains the largest amount of information. Where u is the eigenvector of each feature.

$$Pc_{i}=u_{1i}Fe_{1}+u_{2i}Fe_{2}+\cdots u_{pi}Fe_{n}$$ (3)

The main steps of principal component analysis are:

(1) Decentralize, get the average of all features, and then subtract its own mean from each feature for all samples, as shown in Equation 4.

$$Fe_{n}-\left(\frac{1}{M}\sum_{i=1}^{M}Fe_{n}^{i}\right)$$ (4)

Where $Fe_{n}$ is the feature, n is the feature dimension, and M is the number of samples. In this paper, n = 4, M = 400.

(2) According to Equations 5, 6, calculate the covariance matrix of M samples under n-dimensional characteristics. Where C is the covariance matrix, $\text{cov}\left(Fe_{n},Fe_{n}\right)$ is the covariance, and is the average value of the n-dimensional feature sample.

$$C=\left[\begin{array}{ccc} \text{cov}\left(Fe_{1},Fe_{1}\right) & \cdots & \text{cov}\left(Fe_{1},Fe_{n}\right)\\ \vdots & \ddots & \vdots \\ \text{cov}\left(Fe_{n},Fe_{1}\right) & \cdots & \text{cov}\left(Fe_{n},Fe_{n}\right) \end{array}\right]$$ (5)

$$\text{cov}\left(Fe_{n},Fe_{n}\right)=\frac{\sum_{i=1}^{M}\left(Fe_{n}^{i}-\bar{Fe_{n}}\right)\left(Fe_{n}^{i}-\bar{Fe_{n}}\right)}{M-1}$$ (6)

(3) By deriving Equations 7, 8, find the eigenvalues of the covariance matrix and their corresponding orthogonalized unit eigenvectors

Cu=λu (7)

$$\begin{array}{l} \left[\begin{array}{l} Pc_{1}^{i}\\ Pc_{2}^{i}\\ \cdot \\ \cdot \\ \cdot \\ Pc_{m}^{i} \end{array}\right]=\left[\begin{array}{l} u_{1}^{T}\cdot {\left(Fe_{1}^{i},Fe_{2}^{i},\ldots ,Fe_{n}^{i}\right)}^{T}\\ u_{2}^{T}\cdot {\left(Fe_{1}^{i},Fe_{2}^{i},\ldots ,Fe_{n}^{i}\right)}^{T}\\ \cdot \\ \cdot \\ u_{m}^{T}\cdot \left(Fe_{1}^{i},Fe_{2}^{i},\ldots ,Fe_{n}^{i}\right) \end{array}\right]\\ i=1,2,\ldots ,M. \end{array}$$ (8)

**FIGURE 13:**
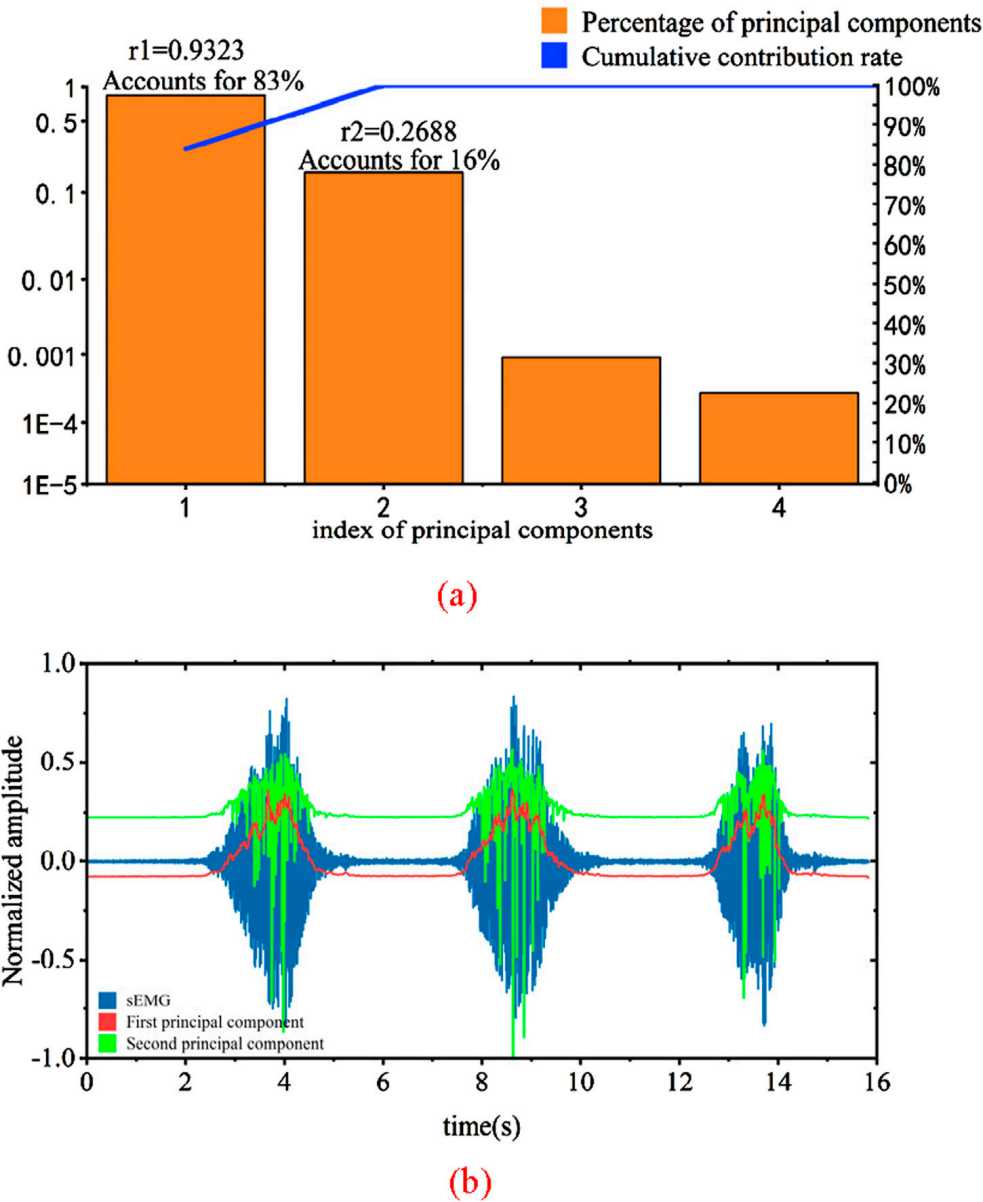
Principal component analysis results and input signal graph. **(a)** Results of principle component analysis **(b)** Principal component of sEMG signals.

After the screening of features and muscles above, it is a set of feature vectors. Because the calculation is more complicated, this feature value will be changed into a set of dimension feature vectors after principal component analysis in this paper. Figure 13 shows the principal component analysis results and principal component diagram of a set of EMG data measured in knee extension experiment after extracting characteristic values and then undergoing PCA analysis.

In Figure 13a, the bar chart represents the percentage of variance for each principal component in relation to the total variance, while the line indicates the cumulative contribution rate. It can be observed that the variances of the third and fourth principal components approach zero, and thus, these two components are not selected. The variance contribution of the first principal component is 83%, and the cumulative contribution rate of the first and second principal components is as high as 99%. Therefore, the first and second principal components are chosen as the feature principal components.

By projecting the original features onto the selected principal component feature vectors, the newly obtained 2-dimensional features after dimension reduction are shown in Figure 13b. The correlation coefficients between these two features and their corresponding joint torque are calculated as r1 = 0.9323 and r2 = 0.2688. A higher correlation coefficient indicates a stronger ability of the input signal to represent force changes. It can be seen from the graph that the performance of the first principal component is the strongest, consistent with the earlier analysis. Therefore, the obtained two-dimensional array can effectively reflect the information of the original data. PCA dimensionality reduction significantly reduces computational burden and enhances the real-time capability of the system.

## 4 Regression learning of human joint interaction force signal based on discrete BP neural network

In this section, a backpropagation neural network (BPNN) was employed to identify joint interaction torques. The model inputs were sEMG signals, standardized to the range [−1, 1]. The output torque was constrained within a biomechanically plausible range [−30, 30] to avoid unphysiological values. To prevent overfitting, L2 regularization was applied to constrain network weights, enhancing generalization. The sEMG signals were sampled at 1,000 Hz with a time step of 1 ms. To ensure responsiveness to dynamic motions, input data underwent sliding window processing: a window length of 200 ms (200 data points) and a 50% overlap rate. Mean squared error (MSE) served as the loss function during training, with performance evaluated on a validation set. Training was capped at 1,000 epochs, and an early stopping strategy halted training if validation error plateaued for 50 consecutive epochs. A learning rate adjustment policy gradually reduced the rate as validation error stagnated until convergence. These strategies ensured model stability, reliability, and improved identification accuracy.

### 4.1 Discrete data collection of knee joint interactive torque regression

**FIGURE 14:**
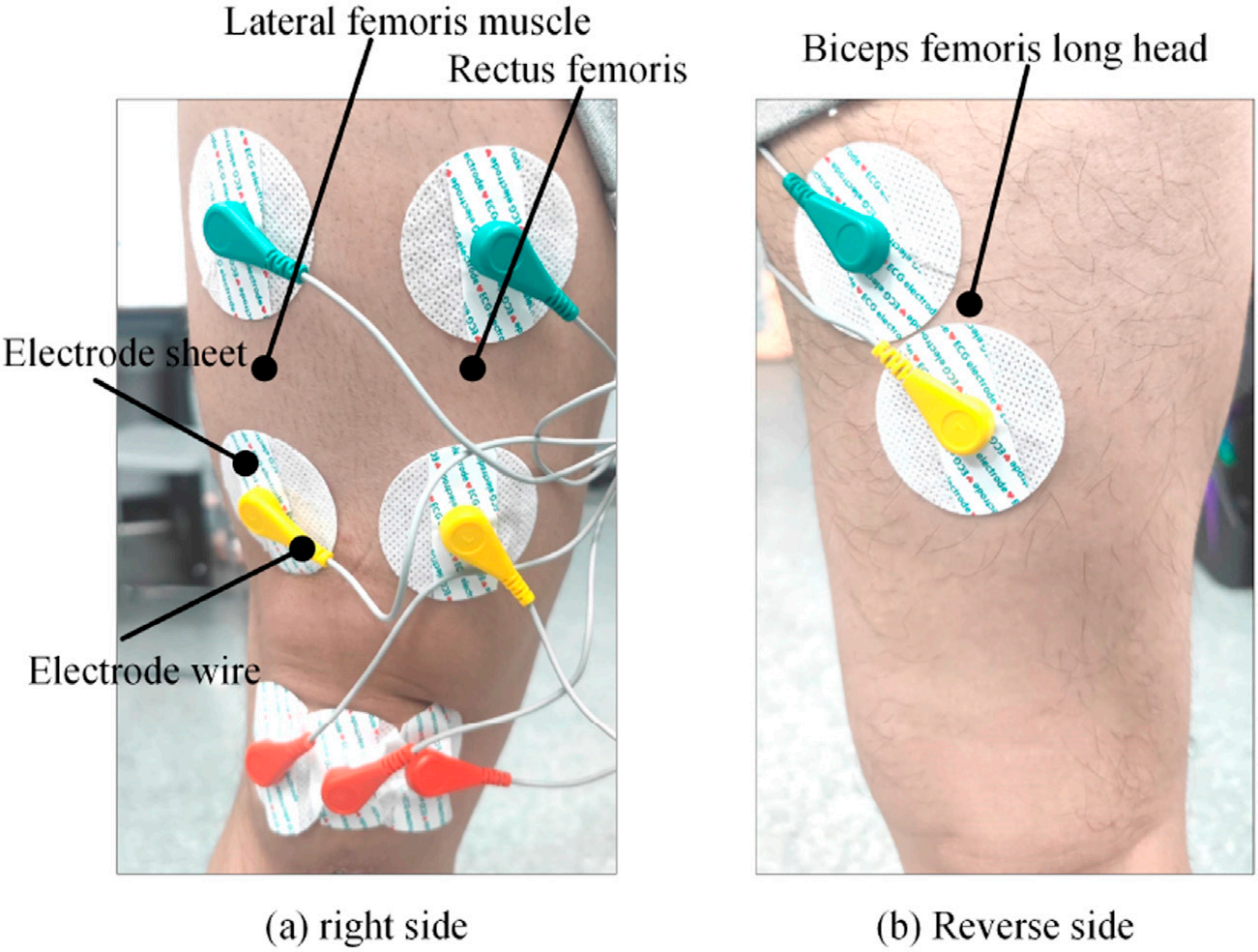
Position of electrode patch. **(a)** Right side **(b)** Reverse side.

The purpose of this experiment is to collect 3-channel sEMG signals and corresponding interaction torque data under different joint angles. The collected data, optimized and paired with the corresponding interaction torque values at each moment, form a dataset used as both the training and testing sets for the interaction regression model proposed in this study. The target variable in the estimation model is the joint interaction torque, and the actual joint torque is obtained based on the static torque sensor installed on the experimental platform. Figure 14 shows a schematic diagram of the electrode placement on the skin.

**FIGURE 15:**
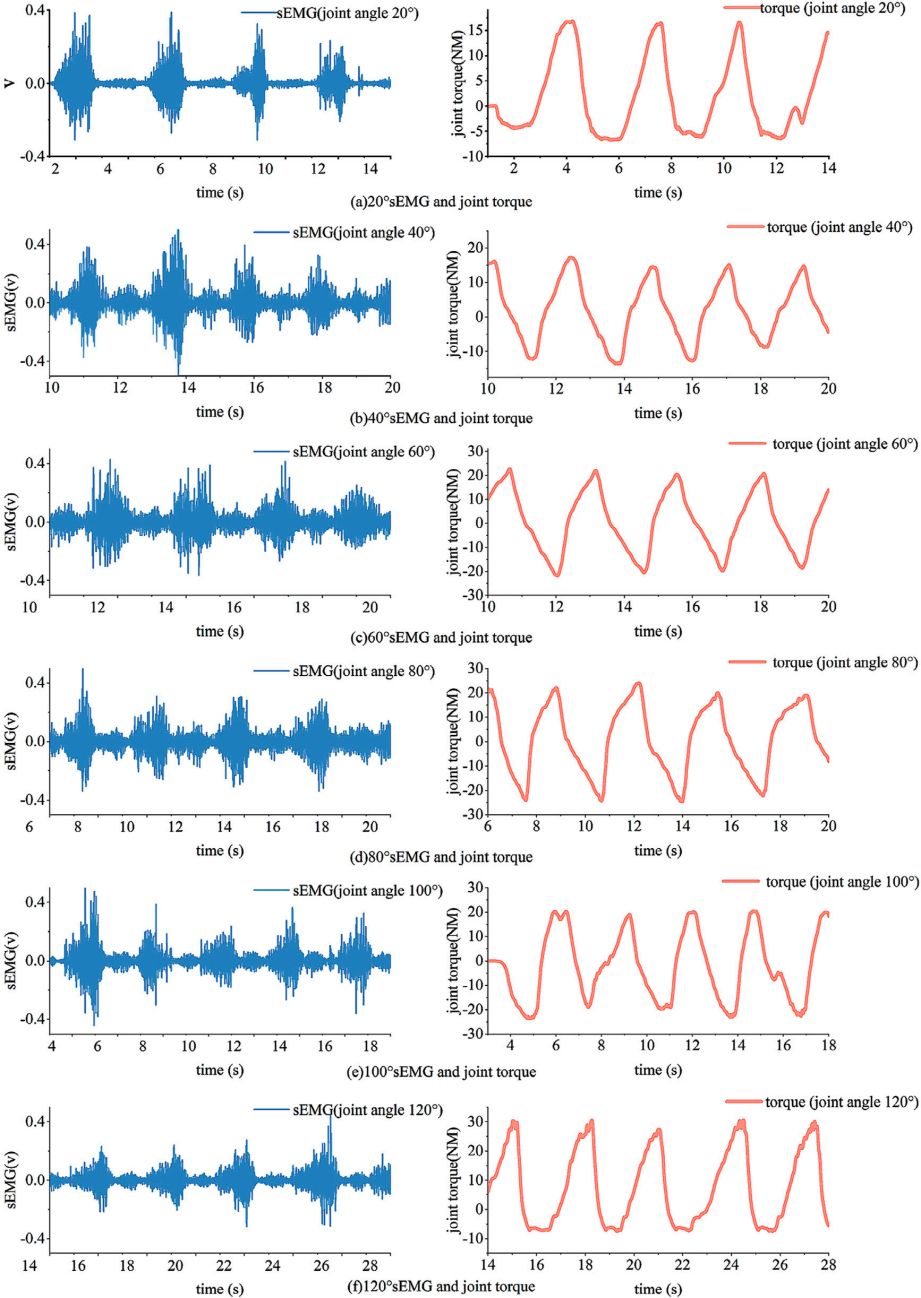
Example of data collection results. **(a)** 20 s EMG and joint torque **(b)** 40 s EMG and joint torque **(c)** 60 s EMG and joint torque **(d)** 80 s EMG and joint torque **(e)** 100 s EMG and joint torque **(f)** 120 s EMG and joint torque.

This experiment collected a total of 12 sets of regression data for knee joint interaction torque under different joint angles. For the needs of training and testing the model, two sets of data were collected in each experiment. Due to the flexibility of the human body when fixed in the device, there is still a range of motion of about 10°. Therefore, the participants adjusted the knee joint fixation device in intervals of 10°, starting from 20° and ending at 130° in each experiment. The partial results of the data collection for the rectus femoris EMG signals and their interaction torque are illustrated in Figure 15.

### 4.2 Discrete joint torque regression algorithm based on BPNN

Based on the previous muscle selection, feature extraction, and dimensionality reduction, the size of a single sample input is determined to be 1 × 7. Therefore, the number of nodes in the input layer of the BP neural network is set to 7. The number of nodes in the hidden layer has a significant impact on the predictive performance of the neural network. If the number of hidden layer nodes is too small, the network may not be trainable, or its performance may be poor. If the number is too large, it can reduce the system error of the network, but on the one hand, it prolongs the training time of the network, and on the other hand, it may lead to the network getting stuck in local minima and fail to reach the optimum point. Overfitting is also a potential issue during training. Currently, the Equation 9 is commonly used to determine the range of the number of hidden layer nodes, and multiple experiments are needed to find the optimal number of nodes.

$$m\leq \sqrt{n+l}+\alpha$$ (9)

In the formula, m is the number of nodes in the hidden layer, n is the number of nodes in the input layer, l is the number of nodes in the output layer, and α is a constant in the range of 0–10. According to Equation 10, node numbers can be chosen as 2, 3, 4, 11, 12, respectively, for model training, with the test results on the test set used as a basis for comparison. Here, root mean square error (RMSE) is introduced to assess the accuracy of the lower limb joint angle prediction results. In the formula, $T_{p}$ represents the predicted joint interaction torque, and $T_{r}$ represents the actual joint interaction torque.

$$\text{RMSE}=\sqrt{\frac{1}{n}\sum {\left(T_{p}-T_{r}\right)}^{2}}$$ (10)

**FIGURE 16:**
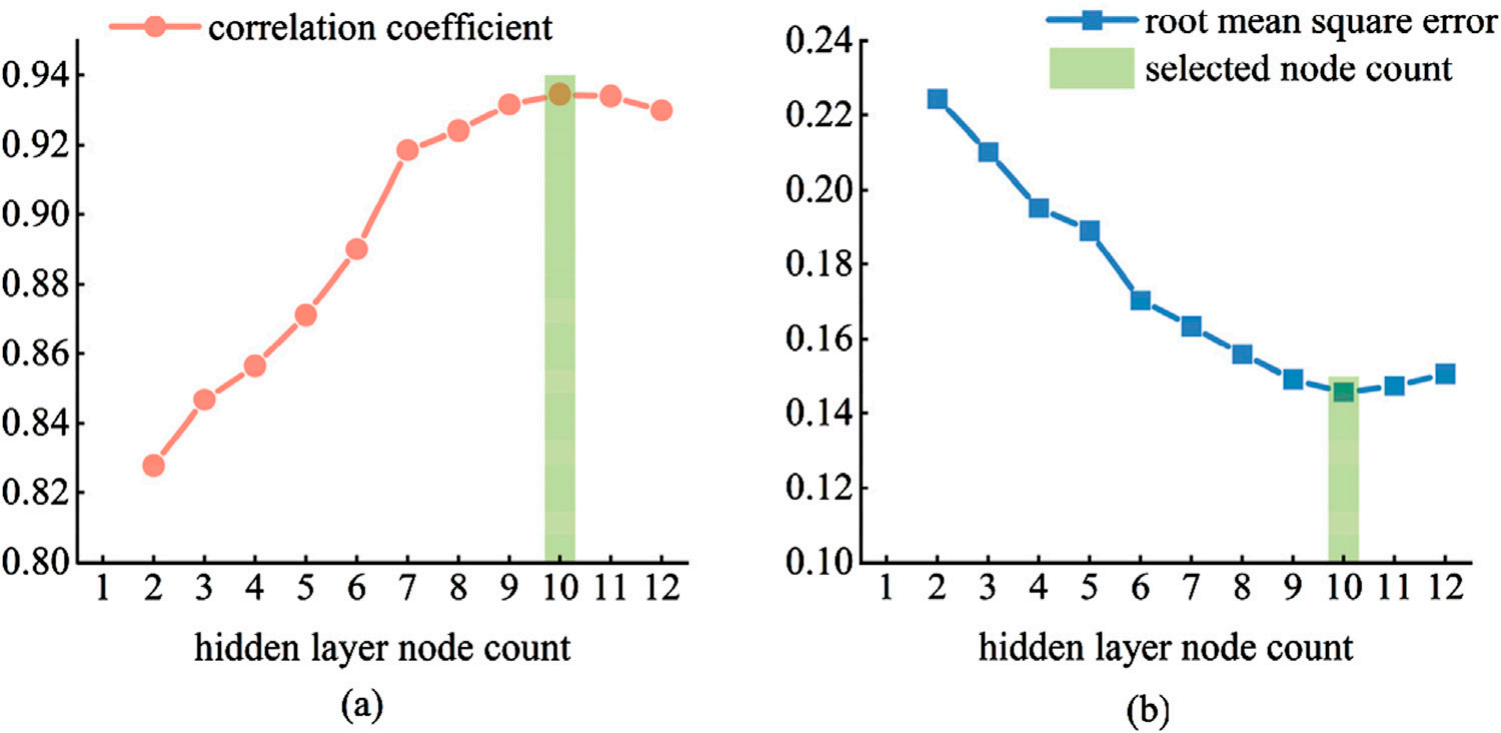
Selection of the number of hidden layer nodes. **(a)** correlation coefficient **(b)** root mean square error.

As shown in Figure 16, initially, with the continuous increase in the number of nodes, the correlation coefficient between the prediction results on the test set and the actual values shows a positive correlation, while the RMSE exhibits a negative correlation. As the number of nodes exceeds 10, the predictive performance of the model begins to decline, with a decrease in the correlation coefficient and an increase in RMSE. This is because, with the increase in the number of nodes in the hidden layer, the model may experience overfitting. Therefore, the final decision is to set the number of nodes in the hidden layer to 10.

## 5 Test verification and analysis

### 5.1 Neural network training and its effect evaluation

**FIGURE 17:**
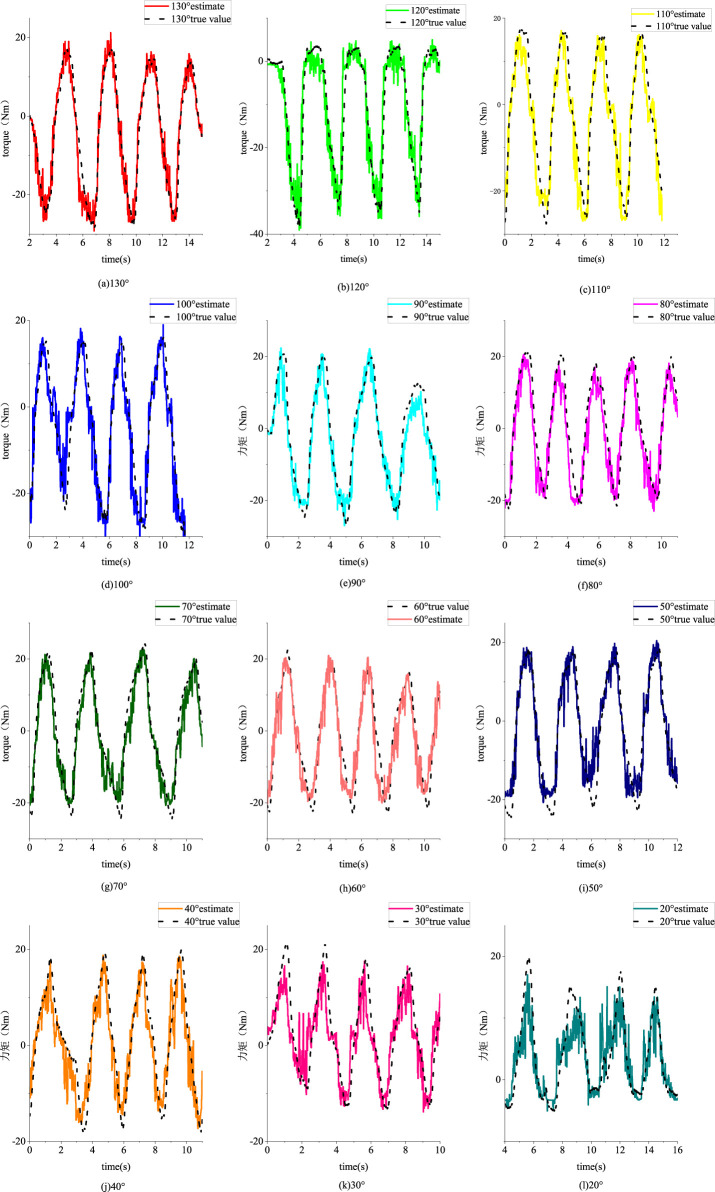
Fitting effect of test sets from different angles. **(a)** 130° **(b)** 120° **(c)** 110° **(d)** 100° **(e)** 90° **(f)** 80° **(g)** 70° **(h)** 60° **(i)** 50° **(j)** 40° **(k)** 30° **(l)** 20°.

Twelve different angle experiments were conducted, and data from each angle were collected in two sets, resulting in a total of 24 sets of experimental data. After preprocessing in MATLAB, the sEMG data were windowed, and four features were calculated for each of the quadriceps femoris, vastus lateralis, and biceps femoris long head muscles. Through PCA, these muscle features were reduced to two dimensions. The resulting features, along with the joint angles, formed a feature matrix of size 1 × 7. The joint interaction torque data were low-pass filtered and compiled into a target matrix. Each set of experimental data at different angles was combined as a training set for building the EMG-joint torque model, while the other set was used as a test set for model evaluation. Figure 17 shows the fitting results using a BP neural network for the joint interaction torque from the 12 different angle test sets.

This paper will use Root Mean Square Error (RMSE), Coefficient of Determination ( $R^{2}$ ), and Pearson Correlation Coefficient ( r ) as evaluation metrics for the regression model. RMSE is used to represent the average error between the model’s predicted values and the true values, with lower values indicating better fitting performance. $R^{2}$ reflects how close the model’s predicted values are to the variance of the true values, with values closer to 1 indicating better alignment with the true values. Pearson Correlation Coefficient R is used to compare the similarity between the predicted joint interaction torque and the actual joint interaction torque. The Equation 11 for calculating $R^{2}$ is as follows:

$$R^{2}=1-\frac{\sum_{i=1}^{n}{\left(T_{p}-T_{r}\right)}^{2}}{\sum_{i=1}^{n}{\left(T_{r}-\bar{T}\right)}^{2}}$$ (11)

Where $T_{p}$ is the predicted value, $T_{r}$ is the actual value, $\bar{T}$ is the average of all the actual values, and n is the total number of samples.

The regression performance under different Angle test sets is shown in Table 3. Under each index, the performance of models from different angles is good. The mean square error of all experiments is 0.1502, the mean coefficient of determination is 0.8616, and the mean coefficient of correlation is 0.9365.

**TABLE 3:** Regression evaluation indicators of test set models from different perspectives.

Experimental angle	*RMSE*	*R* ^2^	*r*
20°	0.1425	0.8533	0.9239
30°	0.1338	0.8797	0.9453
40°	0.1527	0.8813	0.9530
50°	0.1690	0.8665	0.9346
60°	0.1445	0.8722	0.9545
70°	0.1710	0.8325	0.9426
80°	0.1559	0.8643	0.9552
90°	0.1713	0.8322	0.9243
100°	0.1407	0.8793	0.9500
110°	0.1440	0.8895	0.9271
120°	0.1657	0.8814	0.9007
130°	0.1120	0.8069	0.9268
Mean value	0.1502	0.8616	0.9365

### 5.2 Actual joint interaction torque extraction

Based on the learning results of the neural network mentioned above, practical tests were conducted for predicting joint interaction torques during actual gait. Since the torque learning in this paper is in a discrete form, meaning that the parameters of the neural network are different for different angles, the obtained numerical values of human joint interaction torque also exhibit interval variability. As the subsequent human joint interaction torque will be introduced as a control target, it needs to be processed into continuous data. This paper mainly uses Kalman filtering and low-pass filtering to obtain the learning results of human joint interaction torque.

**FIGURE 18:**
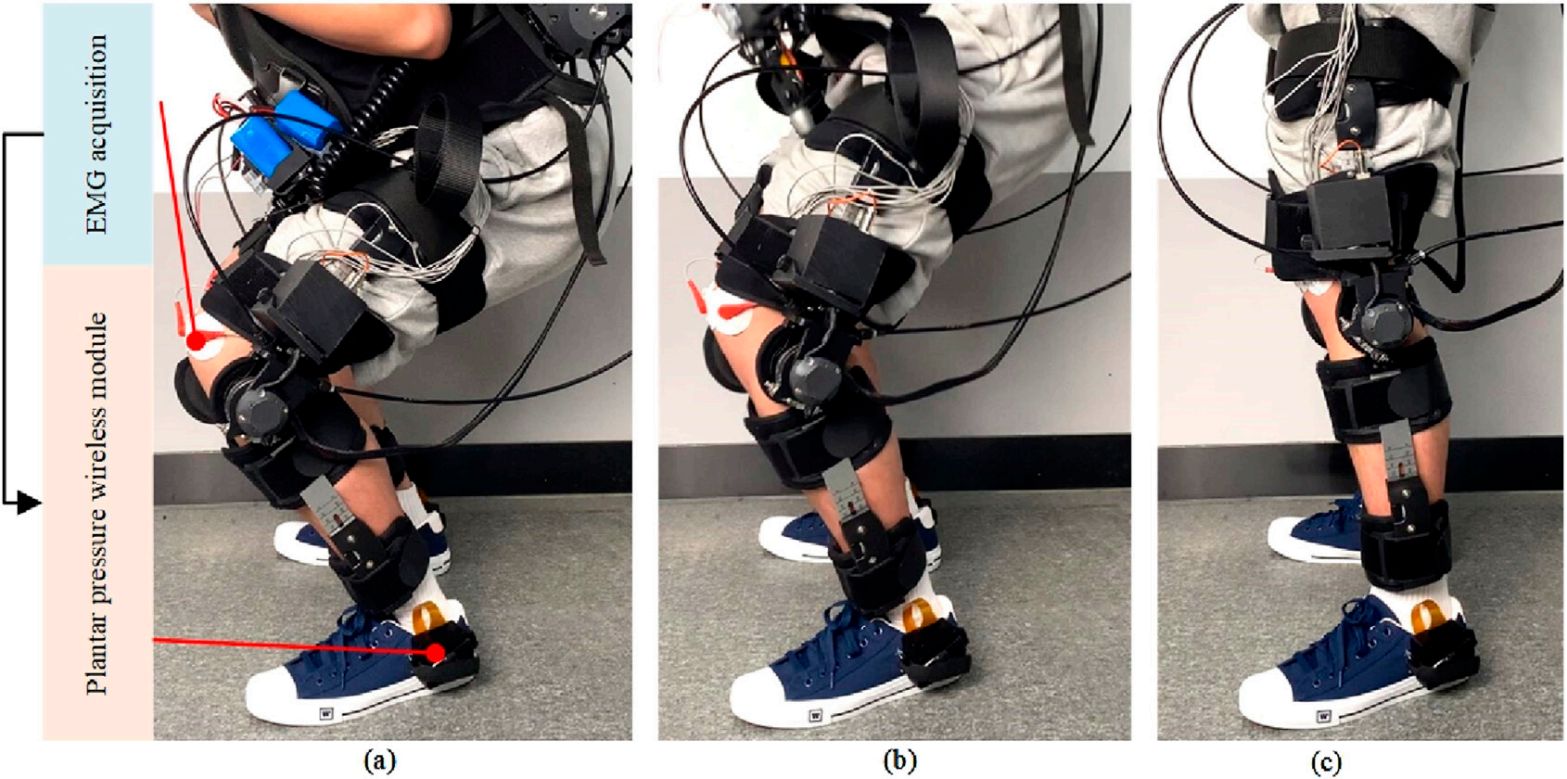
Experimental process. **(a)** Stand down **(b)** Stand up **(c)** Stand.

**FIGURE 19:**
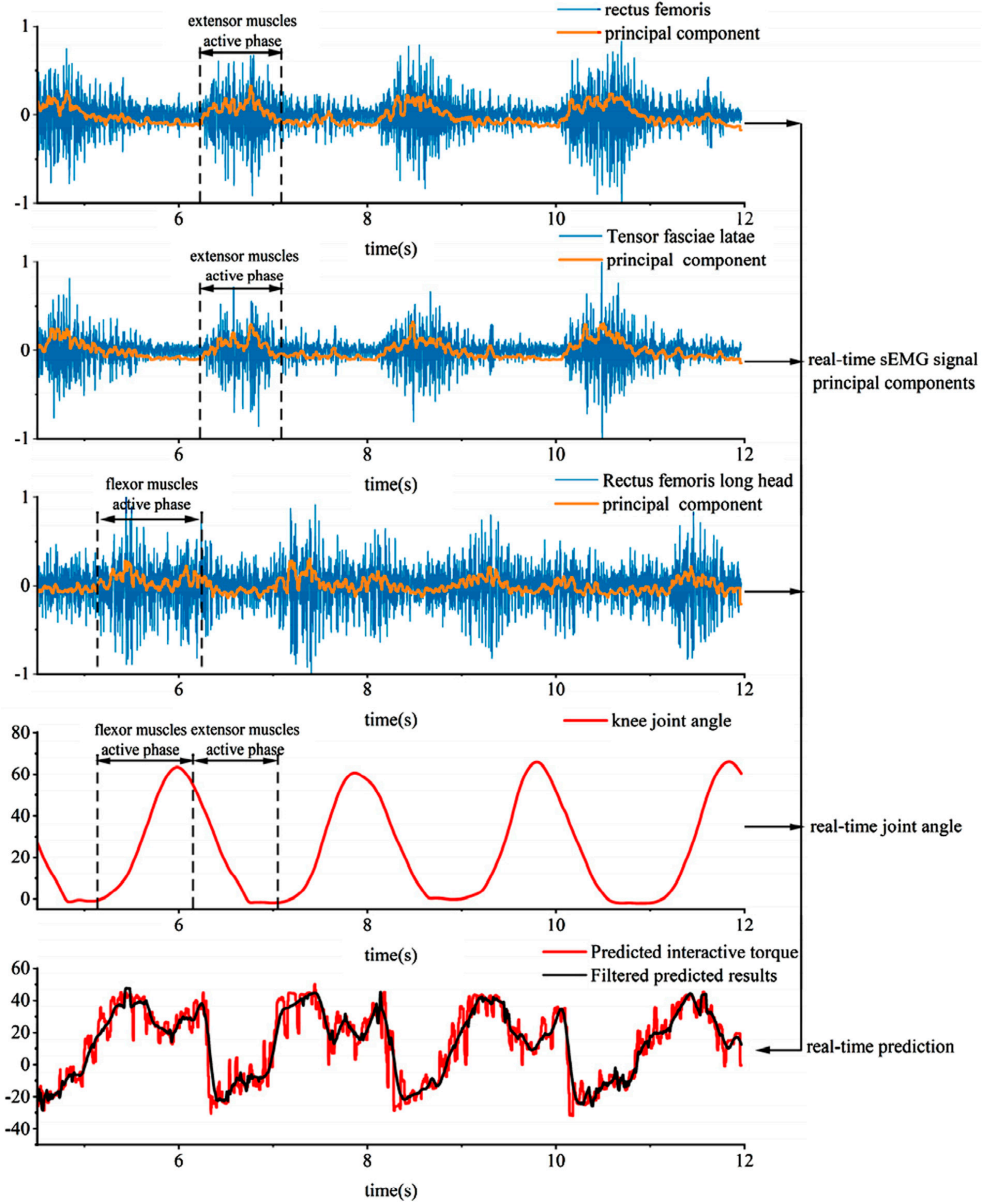
Test results of actual gait.

As shown in Figure 18, the experimental process for collecting human joint interaction torque involves the subject wearing an exoskeleton robot and performing squatting exercises. Since there is no swing phase during squatting, both joints of the exoskeleton robot are in a supporting phase. The test results in Figure 19 include experimental muscle signal data and knee joint angle data during movement:

The experiment mainly focused on the continuous collection of EMG signals from the quadriceps femoris, vastus lateralis, and biceps femoris muscles of the thigh during motion. The first principal component obtained in real-time through PCA dimensionality reduction of the features is representative of the motion characteristics, as analyzed through the characteristic curve.

Since the aforementioned neural network feature learning is conducted discretely, meaning that the EMG signals are segmented into units of ten degrees within the joint motion range, separate neural networks are established based on the joint interaction torque curve. Due to the discretized nature of setting the learning intervals for the network, the obtained curve exhibits certain unstable and jumping patterns, as shown in the interaction force prediction results in Figure 19. Applying Kalman and low-pass filtering to the above results can achieve coherent signal collection and extraction for predicting interaction forces.

**FIGURE 20:**
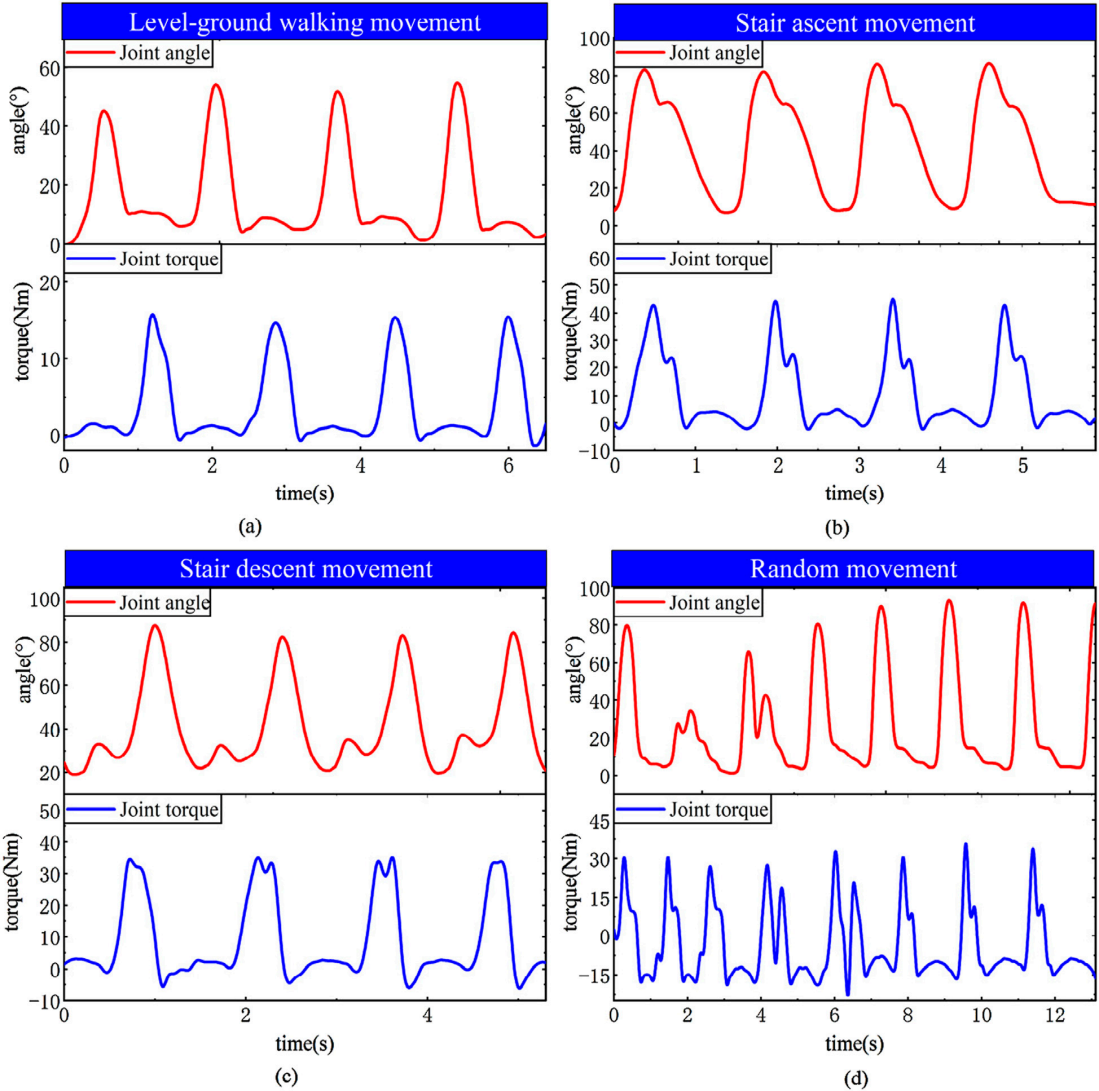
EMG regression learning curves across motion states. **(a)** Level-ground walking **(b)** Stair ascent **(c)** Stair descent **(d)** Random.

The principal component features of muscles can reflect muscle activity. Comparing the principal components of various muscles with knee joint angles in the experimental results reveals that during knee flexion, the flexor muscles are more active, while during knee extension, the extensor muscles are more active. Moreover, based on the predicted interaction torque results, it can be observed that during knee flexion, the interaction torque value is positive, and the flexor torque is relatively large compared to the extensor torque. These experimental results overall conform to the objective laws. To evaluate the joint torque extraction performance of the proposed method during actual movements (Figure 20), tests were conducted using a wearable device during four motion modes: level walking, stair ascent, stair descent, and random motion. The torque curves revealed peak torque angles of 9°–11° (level walking), 69°–74° (stair ascent), and 31°–38° (stair descent), corresponding to the maximum load-bearing moments in the stance phase. Torque magnitudes exhibited clear distinctions between the stance phase (higher amplitude) and swing phase (lower amplitude). In random motion, the torque variation frequency synchronized precisely with angular kinematics, demonstrating consistency between extracted torque values and physical motion dynamics. These results confirm the method’s accuracy in torque quantification across diverse motion states.

## 6 Conclusion

This paper explores the discrete extraction technology of human joint interaction torque based on EMG acquisition. A method utilizing a discrete backpropagation neural network was implemented to conduct regression learning of human joint interaction force signals. By training the neural network, the relationship between input characteristics and target torque was established, enabling torque prediction. Through this study, a discrete prediction technology for human joint interaction torque based on EMG acquisition was developed. This technology contributes to a deeper understanding of the relationship between muscle activity and joint motion while providing a feasible method for extracting human joint torque.

## Data Availability

The raw data supporting the conclusions of this article will be made available by the authors, without undue reservation.

## Abbreviations

BPNN, Backpropagation neural network; EMG, Electromyography; HITL, Human-in-the-loop; LSTM, Long short-term memory; MAV, Mean absolute value; MSE, Mean squared error; PCA, Principal component analysis algorithm; RBF, Radial basis function neural network; RMS, Root mean square; RMSE, Root mean square error; sEMG, Surface electromyography; SPGP, Sparse gaussian processes; VAR, Variance; WL, Wavelength; ZC, Zero crossing count.
